# Patients’ ability to read and understand dosing instructions of their own medicines – a cross sectional study in a hospital and community pharmacy setting

**DOI:** 10.1186/s12913-018-3252-1

**Published:** 2018-06-07

**Authors:** M. G. C. A. Manchanayake, G. R. W. S. K. Bandara, N. R. Samaranayake

**Affiliations:** 1 Ministry of Health, Colombo, Sri Lanka; 2 Department of Allied Health Sciences, Faculty of Medical Sciences, University of Sri Jayewardenepura, Gangodawila, Nugegoda Sri Lanka

**Keywords:** Dosing instructions, Dispensing labels, Pharmacists, Readability, Completeness, Comprehensibility, Medication safety, Medication errors

## Abstract

**Background:**

Poor communication of medicines information to patients may cause medication errors. We assessed the completeness and readability of dosing instructions provided by pharmacists on dispensing labels and knowledge among patients on dosing instructions of their medicines.

**Methods:**

A cross sectional study was conducted in a selected teaching hospital, and a community pharmacy, among 800 patients selected through a systematic sampling method, during a period of 2 months. Completeness of dosing instructions were assessed against a checklist. Patients were asked to read dosing instructions to assess readability. Patient knowledge on dosing instructions were determined through a questionnaire. Completeness, readability and knowledge were scored out of 10 for each dispensing label.

**Results:**

A total of 1200 and 1372 dispensing labels were assessed in the hospital and community settings respectively. The median score out of 10, for completeness, readability and patient knowledge of dosing instructions were 6.7, 8.3 and 7.5 respectively in the hospital, and 7.5, 8.0 and 7.5 respectively in the community. Only a few dispensing labels had the route of administration (hospital, 0.5%; community, 0.8%) and the duration of treatment written (hospital, 0.25%; community, 0.65%) on them. Name (hospital, 48%; community, 27.3%) and strength (hospital, 40.2%; community, 36.6%) of medicines on dispensing labels were frequently misread. In both settings, readability scores significantly differed with education level (*P* < 0.001).

**Conclusions:**

Some important dosing instructions were missing in dispensing labels. Readability of dosing instructions by patients was also not 100% and differed by their education level. Pharmacists did not adhere to a standard procedure in providing dosing instructions leading to communication gaps with patients. Hence we recommend the development of a standard procedure to provide complete, clear and simple dosing instructions to patients, and continuous training for pharmacists on proper communication of dosing instructions to patients.

**Electronic supplementary material:**

The online version of this article (10.1186/s12913-018-3252-1) contains supplementary material, which is available to authorized users.

## Background

Providing information to patients about their medication is a fundamental responsibility of the pharmacist. The information provided needs to be comprehensive, readable and understandable for maximum benefit. Among all medicines information that needs to be communicated, the patient must at least know the dosing instructions for each medication they are taking. Failing to effectively communicate dosing instructions such as, the name, strength, frequency, duration, route of administration and important adverse effects of the medication may be detrimental to the patient.

Poor knowledge about their own medications among patients could result in misuse [1] and poor compliance [2], both of which will negatively impact medication safety. If patients are unaware of their medication names or strengths, they may consume the wrong medication, wrong strength or even duplicate medicines with different brand names. They will not be able to check if medicines they buy are appropriate, or communicate to other health professionals about the medications they use when needed. If dosing instructions are incomplete, misread or misunderstood, patients may consume medication at a wrong time, duration or even route [3]. In fact, we have come across instance where patients have swallowed suppositories and respules indicating the impact of poor medicine knowledge, on medication safety. The magnitude of this problem will increase among older patients as most take a number of medicines at a time [4].

Poor knowledge among patients about their medication could also affect the cost of healthcare in a country [5]. Sub-therapeutic outcomes or toxicities that result as a consequence of improper administration of medicines may prolong hospitalization or increase re-admissions [6]. Simply, outpatient healthcare will completely collapse if patients who are correctly diagnosed with carefully planned treatment regimens, do not take their medication as prescribed. Hence the pharmacists’ ability to effectively communicate medicines information to patients is a topic of national importance.

Studies have assessed the level of communicating dosing instructions to patients, especially the effectiveness of communication, and factors that affect proper communication, at observational and interventional levels [7, 8]. The impact of factors such as, patient literacy, number of medicines dispensed, format and organization of the medicines label, complexity of dosing instructions, precision of writing dosing instructions and the use of icons, graphics and pictograms, on communication of medicines information have been documented in the literature [9–11]. However, a considerable gap was clearly evident. Most published studies assessed patients’ readability and comprehensibility of medicines information using mock dispensing labels and not their own medications [4, 5, 12–19]. A thorough literature survey by the authors revealed that, studies that assessed these characteristics in the real-world setting were very limited. It is also important to note that, a patient’s ability to read a simulated dispensing label may not be completely indicative of the readability and comprehensibility of their own medications labels [20]. Among the few reported, is a considerably large study conducted by Athuruliya et al.*,* [21] where completeness and understandability of dosing instructions given with dispensing labels were assessed in a house-hold survey. However, understandability of dosing instructions were assessed as a simple yes/no question without using objective criteria which the authors highlight as a limitation [21]. Unaka et al., [12] assessed written instructions provided at discharge by a hospital medication service but the study was limited to paediatric medication charts. Moreover readability and understandability of written instructions were assessed retrospectively using the Fry Readability Scale (FRS) and Patient Education Materials Assessment Tool (PEMAT) tools respectively, and not by directly assessing patients ability [12]. Law et al.*,* [22] conducted a prospective, exploratory study on readability and understandability of dispensing labels in the real world setting by interviewing 179 patients which is relatively a smaller sample when considering cross sectional studies [22].

With an aim to bridge these gaps, we set out to study the completeness, readability and overall knowledge among patients of written dosing instructions provided by pharmacists on dispensing labels in a ‘real world’ hospital and community setting.

## Methods

### Study design and settings

A descriptive, cross sectional and prospective study was conducted among patients attending out-patients pharmacies in a selected teaching hospital (study hospital pharmacies) and a selected community pharmacy (study community pharmacy) in the Colombo district. Although, the study settings were selected through convenience sampling, the study hospital is one of the main tertiary care hospitals in Sri Lanka, and the community pharmacy is an outlet of the only state owned pharmacy chain in Sri Lanka.

The study hospital is a tertiary care hospital with a bed strength of 1099 and approximately 40 different types of functioning outpatient clinics. There are three out-patient pharmacies to serve patients who attend these clinics. Medicines are dispensed to around 2100 clinic and other out-patients through these pharmacies per day. The study community pharmacy is one outlet of the state owned community pharmacy chain, and serves around 600 patients a day.

In Sri Lanka, essential dosing instructions are provided to patients by the pharmacist in written form based on the prescription provided by the prescriber. The common practice is to have pre-semi-printed labels on medicine envelopes which will be used for packing medicines. The semi-printed label includes, typed sentences of dosing instructions, with blank spaces to fill the information which varies with the type of medication. Sometimes, the printed forms are not available and pharmacists use the face of the blank envelope to write dosing instructions.

### Study participants

Participants were included in the study if, they were patients or caregivers over 18 years of age, who could speak and understand Sinhala and/or English language, and were dispensed at least one medication from study pharmacies. Those who were illiterate, disabled, diagnosed with psychiatric disorders, dispensed only surgical/medical devices, or dispensed only external preparations were not selected.

### Sample size calculation and sample selection

The number of participants were calculated separately for hospital and community settings using an online sample size calculator (Raosoft. Inc) and considering 95% confidence level, 5% significance, and a response distribution of 50%. A minimum sample size of 384 participants was calculated for each setting, and 400 participants were selected from each setting (*N* = 800) after anticipating a drop-out rate of 10%.

A systematic random sampling technique was used to select study participants. Every 5th patient/caregiver who attended the study setting during a specified time on weekdays, and fulfilled the inclusion criteria, were selected for the study until the minimum sample size (400 from each setting) was achieved. If a participant did not consent to take part or did not fulfill the inclusion criteria, the next patient according to the systematic sampling technique was approached.

### Data collection and scoring procedure

Data were collected by two research pharmacists during a period of 2 months using a pre-determined data collection form (Additional file 1). The two researchers were trained on the data collection process, and both were engaged in collecting data from one patient at a time to minimize inter-researcher variability in data collection. The two researchers approached the patient to be selected, and explained the study process.

#### Assessing the completeness of written dosing instructions

A list of essential dosing instructions that should essentially be provided with dispensed medication were compiled by researchers using the ‘Guidelines for Dispensing of Medicines Developed by Pharmacy Board of Australia’ [23], Prescription Drug Products Labeling; Medication Guide Requirement [24] and WHO Good Dispensing Practices [25], and the list was further endorsed by three pharmacy experts in Sri Lanka.

The two researchers observed if these essential dosing instructions were written on each dispensing label. A score of one was awarded for each dosing instruction written on the dispensing label, and was summed up to obtain a total score for ‘completeness’ of dosing instructions for each dispensing label/medication. The total scores were converted to a score out of 10, using the following formula for ease of comparison.

$$ \mathrm{Score}\ \mathrm{out}\ \mathrm{of}\ 10\ \mathrm{for}\ \mathrm{completeness}\ \mathrm{of}\kern0.50em \mathrm{dosing}\ \mathrm{instructions}\kern0.5em \mathrm{for}\ \mathrm{a}\ \mathrm{medication}=\frac{\mathrm{Number}\ \mathrm{of}\ \mathrm{correctly}\ \mathrm{written}\ \mathrm{dosing}\ \mathrm{instructions}\ \mathrm{per}\ \mathrm{medication}}{\mathrm{Number}\ \mathrm{of}\ \mathrm{dosing}\ \mathrm{instructions}\ \mathrm{that}\ \mathrm{should}\ \mathrm{be}\ \mathrm{written}\ \mathrm{by}\ \mathrm{the}\ \mathrm{pharmacist}\ \mathrm{for}\ \mathrm{the}\ \mathrm{type}\ \mathrm{of}\ \mathrm{medication}\ \mathrm{dispensed}}\mathrm{x}\ 10 $$

An incomplete dosing instruction included, essential dosing instructions missing in dispensing labels, use of unapproved abbreviations to define medicine names or any other dosing instruction, and use of illegible instructions, difficult for researchers to read or interpret. Both printed and handwritten forms of instructions were considered appropriate and adequate. Even a brand name mentioned with no clear indication of a generic name was considered complete, especially in the case of combination products (E.g. Multivitamins, Omega-3-fatty acids). Special instructions were only considered essential depending on the type of medicine.

#### Assessing the readability of dosing instructions

The researchers asked the patient to read each written or printed dosing instruction on the dispensing label of each medicine. Dosing instructions were categorized as readable if patients were able to correctly and completely read the instructions without any assistance. Each correctly read instruction was awarded a score of one. Incompletely or incorrectly read instructions were scored zero. The total score for ‘readability of dosing instructions’, for each medication, was converted to a score out of 10 using the following formula.

$$ \mathrm{Score}\ \mathrm{out}\ \mathrm{of}\ 10\ \mathrm{for}\ \mathrm{read}\mathrm{a}\mathrm{bility}\ \mathrm{of}\kern0.50em \mathrm{dosing}\ \mathrm{instructions}\ \mathrm{for}\ \mathrm{a}\ \mathrm{medication}=\frac{\mathrm{Number}\ \mathrm{of}\ \mathrm{correctly}\ \mathrm{read}\ \mathrm{dosing}\ \mathrm{instructions}\ \mathrm{per}\ \mathrm{medication}}{\mathrm{Number}\ \mathrm{of}\ \mathrm{dosing}\ \mathrm{instructions}\ \mathrm{written}\ \mathrm{by}\ \mathrm{the}\ \mathrm{pharmacist}\ \mathrm{for}\ \mathrm{the}\ \mathrm{type}\ \mathrm{of}\ \mathrm{medication}\ \mathrm{dispensed}}\mathrm{x}\kern0.5em 10 $$

#### Assessing the knowledge on dosing instructions

Researchers asked patients a predetermined set of questions related to essential dosing instructions of each dispensed medication to verify patients’ understanding on the same. The questionnaire assessed patients knowledge on the name, dosage form, strength, number of units taken at a time, route of administration, frequency of administration, time of taking medicines (before or after meals) related to the medicines they take. The assessment questions were endorsed by three pharmacy experts for appropriateness before data collection. Patients were given a score of one, for each correctly answered question. The total score for ‘knowledge of dosing instruction’ for each medication, was converted to a score out of 10 using the following formula.

$$ \mathrm{Score}\ \mathrm{out}\ \mathrm{of}\ 10\ \mathrm{for}\ \mathrm{knowledge}\ \mathrm{of}\kern0.50em \mathrm{dosing}\ \mathrm{instructions}\ \mathrm{for}\ \mathrm{a}\ \mathrm{medication}=\frac{\mathrm{Number}\ \mathrm{of}\ \mathrm{correctly}\ \mathrm{interpreted}\ \mathrm{dosing}\ \mathrm{instructions}\ \mathrm{per}\ \mathrm{medication}}{\mathrm{Number}\ \mathrm{of}\ \mathrm{dosing}\ \mathrm{instructions}\ \mathrm{that}\ \mathrm{should}\ \mathrm{be}\ \mathrm{written}\ \mathrm{by}\ \mathrm{the}\ \mathrm{pharmacist}\ \mathrm{for}\ \mathrm{the}\ \mathrm{type}\ \mathrm{of}\ \mathrm{medication}\ \mathrm{dispensed}}\mathrm{x}\kern0.5em 10 $$

In addition to, incorrect and incomplete responses and failing to respond to questions, a patient interpreting a medicine strength without units or using incorrect units, and interpreting the duration of medicines as ‘a month’ for a 28 day/4 week supply of medicines were also considered incorrect.

### Statistical analysis

Statistical Package for the Social Sciences (SPSS) version 21 was used for data analysis. The total number of medicines dispensed among study participants was used as the denominator for calculating percentages in each setting. Chi square analysis was used to determine relationships between categorical variables. Mann-Whitney-U test and Kruskal Wallis test were used to compare means. A 5% significance level was used when determining *P* values. Participants with missing data were excluded from the analysis.

### Ethical consideration

Patient identifiers were not used when collecting data, and the data sheets were only accessible to investigators. If a serious medication error was detected, the researchers were trained to contact the principle investigator immediately. The principle investigator, upon verifying the error, was to report to the practicing pharmacist regarding this negligence. Direct communication by researchers regarding the quality of medication dispensing was discouraged.

## Results

A total of 1200 and 1372 dispensing labels were assessed in the hospital and community settings respectively. The demographics of study participants in each setting are shown in Table 1. None were excluded due to missing data. Among the 800 study participants, 34 (4.3%) were care givers. Most participants were in the age group 51–70 (51.3%) and most had come to refill prescriptions (*N* = 596, 74.5%). Mode for the number of medicines dispensed to a patient was three in the hospital pharmacy, and two in the community pharmacy setting.

**Table 1:** Demographics of study participants in the hospital and community settings

	HPS			CPS		
Variable	Total	Men	Women	Total	Men	Women
Gender, N (%)	400 (100)	125 (31.3)	275 (68.7)	400 (100)	177 (44.3)	223 (55.8)
Age groups, N (%)						
18–30	29 (7.3)	15 (12)	14 (5.1)	43 (10.8)	17 (9.6)	26 (11.7)
31–50	98 (24.5)	30 (24)	68 (24.7)	92 (23.0)	33 (18.6)	59 (26.5)
51–70	215 (53.8)	60 (48)	155 (56.4)	195 (48.8)	83 (46.9)	112 (50.2)
> 70	58 (14.5)	20 (16)	38 (13.8)	70 (17.5)	44 (24.9)	26 (11.7)
Education level, N (%)						
Grade 1–5	44 (11)	8 (6.4)	36 (13.1)	10 (2.5)	4 (2.3)	6 (2.7)
Grade 6–10	103 (25.8)	33 (26.4)	70 (25.5)	153 (38.3)	71 (40.1)	82 (36.8)
Up to Ordinary Level only	167 (41.8)	59 (47.2)	108 (39.3)	34 (8.5)	15 (8.5)	19 (8.5)
Up to Advanced Level only	79 (19.8)	23 (18.4)	56 (20.4)	135 (33.8)	56 (31.6)	79 (35.4)
Degree level	6 (0.5)	2 (1.6)	4 (1.5)	27 (6.8)	14 (7.9)	13 (5.8)
Postgraduate	0 (0)	0 (0)	0 (0)	6 (1.5)	2 (1.1)	4 (1.8)
Other (E.g.Diploma)	1 (0.1)	0 (0)	1 (0.4)	35 (8.8)	15 (8.5)	20 (9.0)
Patient/ caregiver, N (%)						
Patient	380 (95)	119 (95.2)	261 (94.9)	386 (96.5)	171 (96.6)	215 (96.4)
Caregiver	20 (5)	6 (4.8)	14 (5.1)	14 (3.5)	6 (3.4)	8 (3.6)
Prescription type, N (%)						
New prescriptions	96 (24)	42 (33.6)	54 (19.6)	108 (27.0)	53 (29.9)	55 (24.7)
Refill prescriptions	304 (76)	83 (66.4)	221 (80.4)	292 (73.0)	124 (70.1)	168 (75.3)
Number of medicines dispensed						
Total number; Mode	1200			1372		
Mode	3			2		
Min – Max	1–9			1–13		

*HPS* Hospital pharmacy setting, *CPS* Community pharmacy setting

Scores for completeness, readability and knowledge of medicines are shown in Table 2. The mean score out of 10 for completeness, readability and the level of knowledge on dosing instructions for each medication dispensed were above six. A sub-analysis of mean scores by type of dosage form is shown in Table 3. It was notable that the mean score for completeness of dosing instructions was low for sublingual tablets (1.9) and dry powder inhaler capsules (3.7) in the hospital setting. Percentages of medicines that had complete, readable and comprehensible dosing instructions are shown in Table 4. Mean scores for completeness of duration and route of administration were low in both settings (< 1%). Readability and comprehensibility of medicine name and strength were also found to be less than 50% in both settings. In contrast to the community setting where most patients were aware of the duration of treatment (95.5%), duration was known for only 37.2% of medicines in the hospital setting (Table 4).

**Table 2:** Descriptive statistics of scores (out of 10) per medication for completeness, readability and knowledge of dosing instructions among study participants

Score out of 10	Completeness^a^			Readability^a^			Comprehension^a^		
	Mean	Median	SD	Mean	Median	SD	Mean	Median	SD
Hospital pharmacy	6.4	6.7	1.4	8.3	8.3	1.8	7.5	7.5	1.3
Community pharmacy	7.3	7.5	0.6	7.9	8.0	1.5	8.0	7.5	1.3

*SD* standard deviation, *HPS* hospital pharmacy setting, *CPS* community pharmacy setting

^a^The number medicines dispensed was used as the denominator

**Table 3:** Mean score (out of 10) per medication for completeness, readability and knowledge by type of dosage form

	Mean Score out of 10					
	Completeness		Readability		Comprehension	
	HPS	CPS	HPS	CPS	HPS	CPS
Dosage form						
Normal release tablet or capsule	6.5	7.3	8.2	7.6	7.4	7.9
Modified release tablet	6.6	7.4	7.6	8.0	7.5	8.1
Subcutaneous injection	3.4	4.9	10.0	8.5	7.1	6.8
Dry powder inhaler capsules	3.7	4.4	8.9	7.5	7.4	6.2
Sublingual tablets	1.9	4.4	10.0	6.2	5.2	5.0
Syrup or suspension	–	5.4	–	8.2	–	6.9
Powders for reconstitution	–	5.2	–	7.5	–	5.4
Others (Lozenges)	–	4.6	–	4.6	–	5.4

**Table 4:** Percentages of medicines that had complete, readable and comprehensible dosing instructions

Type of instructions	% of medicines					
	Completeness^a^		Readability^b^		Comprehensibility^c^	
	HPS	CPS	HPS	CPS	HPS	CPS
Name	72.2	94.7	48.3	27.3	40.2	25.8
Dosage form	63.6	96.4	92.9	97.1	98.0	95.9
Strength	81.7	89.8	40.2	36.7	29.9	23.9
Number of units dispensed	99.7	99.8	99.1	98.1	99.0	97.4
Frequency	98.9	99.7	98.8	97.4	98.9	96.6
Duration	0.3	0.7	66.7	88.9	37.2	95.5
Route of administration	0.6	0.8	85.7	81.8	99.5	99.7
Relationship with meals (for applicable medicines)	97.5	99.6	91.9	94.9	88.2	91.4
Special instructions (for applicable medicines)	50.8	54.0	96.8	100.0	68.8	48.0

The number of medicines was used as the denominator ^a^, ^b^, ^c^ but varied by medicine type and level of completeness of dosing instructions

^a^number of medicines where the relevant dosing instruction was considered to be essential

^b^number of medicines where the relevant dosing instruction was written by the pharmacist

^c^number of medicines where relevant dosing instruction was considered to be essential

The mean scores for readability of dosing instructions (*P* < 0.001) significantly differed among different education levels, in both settings. Knowledge on dosing instruction of medicines differed by education level in the hospital setting (*P* < 0.001). There was a significant relationship between prescription type (new or refill) and readability in the hospital pharmacy setting (*P* < 0.001) but was not seen in the community pharmacy setting (*P* = 0.064). There was a significant relationship between knowledge of dosing instructions and type of prescription in the community pharmacy setting (*P* < 0.001) but not in the hospital pharmacy setting (*P* = 0.149).

## Discussion

Pharmacist are healthcare professionals who are mainly responsible for providing complete, readable and comprehensible medication information to patients. Medication information is expected to be provided in written, verbal or both forms by the pharmacist. This is one of the very few studies that assessed the completeness of dosing instructions, readability and patients’ level of knowledge of dosing instructions of their own medication. Our main findings show that these three parameters scored a mean of above 6 out of 10 in both settings which is appreciable. However, it also means that dosing instructions were not 100% complete, and patients were not able to completely read or understand dosing instructions, thus a serious gap in the communication process. This is also supported by other studies where only 49% of medicines with patients were adequately labeled [21]. Unaka et al., [12] rated paediatric discharge instructions for completeness, readability, and comprehensibility to conclude that instructions were subpar. Similar to results reported by Shrank et al., [9] and O’Hare et al., [19] readability and comprehensibility of dosing instructions significantly varied across different education levels highlighting the need for providing simplified and patient related dosing instructions.

Some important instructions such as the duration and route of administration were frequently missing in dispensing labels. Duration is an important dosing instructions especially in medicines like antibiotics to achieve desired therapeutic outcomes and to avoid antibiotic resistance. It was evident that most patients who attended the hospital, related a medicines duration of 28 days to 1 month in general. Even though the discrepancy is trivial, a medicine free period of 3 days can be detrimental for long term medications such as anti-hypertensives, anti-diabetics, anti-platelets and thrombolytics. Patients’ knowledge on route of administration is also important. Many unpublished cases have been reported frequently on medicines administered by the wrong route resulting in adverse effects or sub-therapeutic effects. Most commonly mis-administered medicines include, dry powder inhaler capsules, and suppositories, both of which have been swallowed. Inappropriate administration of sublingual medicines may result in sub-therapeutic effects but dosing instruction provided for sublingual tablets were noticeably low. Although the causes for missing duration and route of administration was not assessed in this study, it may be due to, missing data on prescriptions, absence of a standard procedure/format by pharmacists to provide duration and route of administration, or even perceptions by pharmacists that these type of information is not important enough to communicate to patients.

Another interesting finding was that patients often found the name and strength of medicines difficult to read. A patient should know, names and strengths of their medicine to avoid, mix-ups in administration, over dosing and duplications, to self-assess if medicines dispensed to them are correct, and to communicate to other health professionals in an emergency such as medicines allergies. It is unethical for patients to be left ignorant about the medicines they are dispensed with.

Readability, and knowledge of dosing instructions were related to education level. Similar findings were also reported by Davis et al., where poorly literate patients found it more difficult to read and understand dosing instruction [5]. This should be a motivation for pharmacists to provide patient-specific counselling to patients depending on their ability to read and comprehend information. The complex nature of the medicine regimens, and age may also have affected readability and comprehensibility. Findings by Barros et al. supports this claims [26]. A study by Nair, K.V. et al.*,* showed that adequate provision of information is essentially needed when dispensing new prescriptions rather than re-fill prescriptions [27]. Prioritizing on detailed counselling for patients with new prescriptions and limiting to essential dosing instructions and re-clarifications for patients with refill prescriptions could also help to control communication barriers due to heavy traffic at dispensing counters.

We propose that patients with new and re-fill prescriptions be separated when providing medicines information, develop an essential and compulsory list of the minimum dosing instructions to be provided with any medication dispensed, promote pharmacists to write in block capitals or preferably have the dosing instructions printed on dispensing labels, and reconfirm with patient by asking them to read the dispensing labels and explain their medicine to the pharmacist before completing the dispensing process. A special emphasis should be given when communicating dosing instructions to, those with functional barriers, the illiterate, and those with mental illnesses.

There is much strength in our study. Our research was carried out among 800 patients, exceeding 2000 dispensing labels at two settings, a hospital and a community pharmacy. The study included three research techniques, a dispensing label review, assessment of patients’ ability to read dispensing labels in the form of an unstructured interview, and their knowledge on dosing instructions using an interviewer administered questionnaire. We also used a prospective research design and direct communication to collect data from patients/caregivers at the point of obtaining their medication which is more reliable. As acknowledged before, using patients own medicine to study these variables added uniqueness to the study.

There are also some limitations that need to be acknowledged. The settings used for study was selected through convenience sampling and hence may not reflect the pattern in the whole country. Although the study was conducted in two settings, there was no option to compare the two, as one was a hospital, the other was a community pharmacy. However it is a good stimulation for healthcare administrators to take on similar multi-centered studies in their mission to improve medication safety. There may also have been other confounding factors that affected the outcomes of this study. Some patients on long term medication may have been knowledgeable about their medicines through experience, hence scoring high on knowledge of dosing instructions. This may not actually reflect the ability to read and understand dosing instruction written on dispensing labels. We excluded patients dispensed with medical devices and local applications such as creams, ointments to minimize complications. However this exclusion was only an attempt to simplify the research process and not to underestimate dosing instructions that needs to be provided for them. Many administration errors could be related to devices and local applications and we hope to tackle these dosage forms in future studies that follow. Readability and knowledge of dosing instructions were correlated with educational level and not health literacy of patients/caregivers. This could be a limitation as most educated patients may be illiterate on health aspects. Language barriers and other functional barriers such as poor eye sight were not controlled and may have affected our findings. Both verbal and written dosing instructions need to be given by a pharmacist but this study was only limited to dosing instructions written on dispensing labels. It must also be acknowledged that the pharmacists’ ability to communicate dosing information to patients was not assessed.

## Conclusions

This study is one of the few studies that directly appraised the quality of dosing instructions communicated to patients, by pharmacists, on their dispensed medicines. It was evident that current practices among pharmacists on providing dosing instructions varied and did not conform to a standard format. Most often it is assumed that patients are able to read and understand dosing instructions provided to them, but this study indicates that some dosing instructions are mis-read or mis-understood. We urge the need for a standard, universal procedure on providing written dosing instructions for patients. We also highlight the importance of providing clear dosing instructions preferably in clear block capitals or in printed form, devoid of abbreviations. Pharmacists should be advised to consider patient demographics such as age, education level, functional and language barriers and personalize the level of details needed when providing standard dosing instructions. It should be made routine procedure for pharmacists to ask patients to read and explain their medication dosing instructions off the dispensing labels, and clarify any doubts and misinterpretations before completing the dispensing process.

## Additional file

Additional file 1: Patients’ data collection sheet (English) in hospital and community pharmacy settings. (PDF 844 kb)

## Abbreviations

ERC: Ethics Review Committee
FRS: Fry Readability Scale
PEMAT: Patient Education Materials Assessment Tool
SPSS: Statistical Package for the Social Sciences
