# Supplementary material for: Patients’ ability to read and understand dosing instructions of their own medicines – a cross sectional study in a hospital and community pharmacy setting
Source: BMC Health Serv Res. 2018 Jun 7;18:425. doi: 10.1186/s12913-018-3252-1 (PMC5992847; doi:10.1186/s12913-018-3252-1)
Supplement: Supplementary file 1 — Patients’ data collection sheet (English) in hospital and community pharmacy settings. (PDF 844 kb) [file 12913_2018_3252_MOESM1_ESM.pdf]

Additional file 1: Patients' data collection sheet (English) in hospital and community pharmacy settings

"ASSESSING THE COMPLETENESS AND THE LEVEL OF COMPREHENSION OF DOSING INSTRUCTIONS PROVIDED WITH DISPENSED MEDICATION AND THE PERCEPTIONS OF PHARMACISTS ON PROVIDING DOSING INSTRUCTIONS IN A HOSPITAL AND A COMMUNITY SETTING"

Socio - demographic data

1. Age: -

   18-30 Years ☐ 31-50Years ☐ 51-70Years ☐ >70Years ☐

2. Gender: - Male ☐

   Female ☐

3. Education level: - Grade 5 or below ☐ Up to O/L ☐

   Up to A/L ☐ Degree ☐

   Postgraduate ☐ Other ☐

4. Assessment of pharmacist level of counselling at your last visit (Score out of 10)

1	2	3	4	5	6	7	8	9	10

5. Observation:

   Medication data

Prescription type - New ☐ Refill ☐

Prescribed drug plan for the patient

Table 01 - *(Details of each medicine prescribed to the patient)*

DRUG No	Generic name	Dosage form	Strength	No of units per dose	Frequency	Duration	Before /after/ with meals	Route of administration	Special instructions
1									
2									
3									
4									
5									
6									
7									
8									
9									
10									

Table 02 **Assessing completeness of written dosing instructions given by the pharmacist.** *(This information will be observed and recorded by researchers)*

A -Generic name
B - Dosage form
C - Strength
D - No of units per dose
E - Route of administration
F -Frequency of administration -
G - Before /After/With meals
H - Duration
I -Special instructions

	Whether pharmacist has written necessary information? Y - Yes I – Incomplete N - No																										
	A			B			C			D			E			F			G			H			I		
	Y	I	N	Y	I	N	Y	I	N	Y	I	N	Y	I	N	Y	I	N	Y	I	N	Y	I	N	Y	I	N
Drug 1.																											
Drug 2.																											
Drug 3.																											
Drug 4.																											
Drug 5.																											
Drug 6.																											
Drug 7.																											
Drug 8.																											
Drug 9.																											
Drug 10 .																											

Y-Yes; I-Incomplete; N-No

Table 03 - Assessing the readability of the dosing instructions by patient.

1) **Can you read following instructions on the label provided by the pharmacist?**

**A**	-Generic name	**E**	- Route of administration
**B**	- Dosage form	**F**	-Frequency of administration -
**C**	- Strength	**G**	**-** Before /After/With meals
**D**	- No of units per dose	**H**	**-** Duration
		**I**	-Special instructions

**Drug**	**Patient readability**									**Total score***
	**A**	**B**	**C**	**D**	**E**	**F**	**G**	**H**	**I**	
Drug 1										
Drug 2										
Drug 3										
Drug 4										
Drug 5										
Drug 6										
Drug 7										
Drug 8										
Drug 9										
Drug 10										

*Each correct answer will be taken as 1 point, incomplete or wrong answer will be taken as 0 point

. Table 04 - Assessing the patient comprehension of the dosing instructions.
[Researcher will ask patient to interpret all dosing instructions for each drug]

02. pecific questions asked;

i. Do you know the name of this drug?
ii. Do you know the form of this drug?
iii. How much of the drug is there in each drug unit?
iv. How many units are you going to administer at one time?
v. What route will you administer this drug from?
vi. How many times a day will you administer this drug?
vii. How will you administer the drugs in relation to meals? Before/after/with meals?
viii. For how long will you be using this drug for?
ix. Any other special instruction given by the pharmacist/or you know regarding this drug?

A -Generic name
B - Dosage form
C - Strength
D - No of units per dose
E - Route of administration
F -Frequency of administration -
G - Before /After/With meals
H - Duration
I -Special instructions

**Drug**	**Patient comprehension**									**Total score***
	**A**	**B**	**C**	**D**	**E**	**F**	**G**	**H**	**I**	
Drug 1										
Drug 2										
Drug 3										
Drug 4										
Drug 5										
Drug 6										
Drug 7										
Drug 8										
Drug 9										
Drug 10										

*Each correct answer will be taken as 1 point, incomplete or wrong answer will be taken as 0 point

3.) Did the pharmacist provide printed information during your last visit to the pharmacy? (Specify drug)

Yes ☐ No ☐

[If yes] , Drug :…………………………………………………….
Drug :…………………………………………………….
Drug :…………………………………………………….

4) Did you run short of the drug at your previous visit, before you complete the recommended duration?

Drug	Yes	No
Drug 1		
Drug 2		
Drug 3		
Drug 4		
Drug 5		
Drug 6		
Drug 7		
Drug 8		
Drug 9		
Drug 10		

5) Did you have extra units of the drug before you visit this clinic?

Drug	Yes	No
Drug 1		
Drug 2		
Drug 3		
Drug 4		
Drug 5		
Drug 6		
Drug 7		
Drug 8		
Drug 9		
Drug 10		
